# RiNeo MR: A mixed reality simulator for newborn life support training

**DOI:** 10.1371/journal.pone.0294914

**Published:** 2023-12-21

**Authors:** Mara Coduri, Andrea Calandrino, Giulia Addiego Mobilio, Maura Casadio, Serena Ricci

**Affiliations:** 1 Department of Informatics, Bioengineering, Robotics and Systems Engineering, University of Genoa, Genoa, Italy; 2 Simulation and Advanced Education Center - SimAv, University of Genoa, Genoa, Italy; 3 Department of Neuroscience, Rehabilitation, Ophtalmology, Genetics, Maternal and Child Health, University of Genoa, Genoa, Italy; 4 Neonatal Intensive Care Unit, IRCCS Giannina Gaslini Institute, Genoa, Italy; Politecnico di Torino, ITALY

## Abstract

Neonatal resuscitation is an uncommon, albeit critical task that is more likely to succeed if performed properly and promptly. In this context, simulation is an appropriate way for training and assessing the abilities of all medical staff involved in delivery room care. Recent studies have shown that learning is enhanced if the simulation experience is realistic and engaging. Hence, Virtual Reality can be beneficial for newborn resuscitation training. However, the difficulty of providing realistic haptic interaction limits its use. To overcome this constraint, we have designed RiNeo MR, a simulator for newborn life support training, combining a sensorized manikin to monitor in real time resuscitation skills, with a Virtual Reality application. The system includes a Virtual Reality headset, Leap Motion to track the user’s hands, sensorized bag valve mask, and manikin to monitor head and mask positioning, ventilation, and chest compression. RiNeo MR can be used in two modalities: 2D to let the trainee practice resuscitation manoeuvres on the physical manikin, while receiving real time feedback; 3D that allows the user to be immersed in a virtual environment and practice in an hospital-like setting. In the 3D mode, virtual and real manikins are overlapped and communicate in real time. Tests on 16 subjects (11 controls without medical expertise and 5 paediatric residents) demonstrated that the simulator is well tolerated in terms of discomfort. Moreover, the simulator is high rated for user experience and system usability, suggesting that RiNeo MR can be a promising tool to improve newborn life support training. RiNeo MR is a proof of concept of a mixed-reality newborn life support simulator that can be a promising tool to spread newborn resuscitation high-quality training among healthcare providers involved in perinatal medicine.

## Introduction

The transition from intra-uterine to extra-uterine life is a crucial moment for all newborns, as this is the instant in which spontaneous breathing starts [1]. Although the cardio-respiratory extrauterine transition usually occurs spontaneously and at least requires simple stimulation and care manoeuvres, it is estimated that 5–10% of newborns need assistance to establish autonomous breathing [2]; approximately 3–6% of term and late preterm babies receives positive-pressure mask ventilation (PPV), while less than 1% receives chest compressions, advanced airway management and intravenous drugs administration [2]. Since the need for assistance is uncommon and cannot always be predicted [3], it is unlikely that healthcare providers are regularly exposed to neonatal resuscitation. Moreover, pregnant women at risk of preterm delivery or pathologic conditions diagnosed prenatally are usually centralised to hub neonatological centres, further contributing to globally reduce providers’ exposure to complicated deliveries [4]. However, resuscitation is more likely to succeed if it is performed properly and at the right time [2, 5]. Indeed, all staff involved in perinatal medicine (paediatricians, gynaecologists, anaesthesiologists, midwives, nurses, and paramedics) should be prepared to provide the lifesaving interventions quickly and efficiently. Currently, only a few specialists in the delivery room masters Newborn Life Support (NLS), typically well trained neonatologists and anaesthesiologists [6] (see S1 Appendix for further information on the NLS algorithm).

Among the possible ways to increase the number of healthcare provider able to deliver NLS, there is simulation, which allows to practice in a riskless and controlled environment [7]. In fact, many studies have shown that an effective NLS training reduces delivery room death rate by 30% [2]. NLS training is typically achieved using manikins [8]; however, there is increasing evidence that learning is enhanced if the simulation experience is realistic and engaging [9]. Indeed, the use of technologies that increase immersivity, such as virtual reality (VR) and augmented reality (AR) is growing [10, 11]. VR provides an immersive experience that promotes practitioner engagement and supports the acquisition of optimal levels of practical expertise in a safe and controlled environment [7].

In the specific context of emergency medicine training and adult first aid, some VR applications have been developed in the last years [12, 13]. These tools can increase the realism of the simulation, but they cannot train and monitor dexterity skills [11]. Indeed, one of the main limitations of VR for medical training, that may affect the learning output and limit its use, includes the difficulty of providing haptic interaction with the real environment [11]. A possible alternative could be Mixed Reality (MR), since it combines the virtual environment with objects in the real world [14], thus enhancing the VR experience. With MR, user interactions (such as grabbing objects or performing actions with them) occur with the real objects, allowing the user to perceive the shapes and sizes of the objects they see in VR [14], as virtual and real objects are overlapped and aligned. Therefore, mixed reality enables the provision of passive haptics, where this term refers to the ability to oppose a tangible passive object, co-located with the virtual object, to the actions of the user in order to enhance the overall immersive experience [15–17].

The use of Extended Reality (XR, acronym that includes VR, AR, and MR [18]) for medical learning is a powerful tool in the clinical training environment, and several adult learning theories supports its use. They include the Constructivist Learning Theory, namely, learning by interacting with the environment; the Situated Learning Theory, promoting the idea that learning should be embedded within cooperative activities; the Embodied Cognition Theory, that is based on the concept that cognition is the result of the relationship between mind and body [19, 20]. Real situations have always been preferred for adult learning, as learners find it difficult to transfer the theoretical knowledge to the related situation. Also, they prefer engaging learning experiences, where they focus on how to improve their performance [21]. MR provides a controlled environment, allowing learners to navigate it and manipulate objects, and to repeat the same task multiple times [19]. Also, participants usually feel more relaxed and spontaneous with respect to traditional manikin-based simulation [20]. Another important point concerns the sense of presence and engagement of XR simulations, which could help achieving learning outcomes, especially in the case of repetitive activities [22, 23]. Furthermore, MR allows to integrate manual tasks within a realistic context where the knowledge would later be applied, and to manipulate variables, thus providing personalized training [19]. Finally, with MR, users can combine multisensory cues (i.e., visual, auditory, and haptic stimuli) to build new knowledge [21]. The involvement of the proprioceptive system during learning in the synthetic environment would deepen learning and recall [20, 22, 23].

Recently, some research groups implemented MR prototypes for adult life support training [12, 24–27]; they combine a non sensorized manikin, either half body or full body, with a Head Mounted Display (HMD), tracking devices (controllers, trackers, data gloves). Tools for paediatric resuscitation training are still limited to few serious games or AR applications [28–30]. Furthermore, to the best of our knowledge, no MR-based NLS tools are available so far, despite its unquestionable value [11, 30].

For this reason, we have designed RiNeo MR, a mixed-reality system combining a newborn manikin, sensors to monitor resuscitation skills, and VR application. The goal of the project was to design and implement a realistic and engaging educational tool for the training and evaluation of neonatal life support skills. The simulator combines a VR application with neonatal manikin and a face mask, both sensorized to monitor head and face mask position, ventilation, and chest compression. After the design and implementation phase, we have tested the simulator on a cohort of paediatric residents and of people without medical expertise, to evaluate its usability.

## Materials and methods

RiNeo MR combines a sensorized newborn manikin and face mask (hardware), with a VR application consisting in 3D scenarios of delivery and operating rooms, and a virtual representation of the manikin (software). The two parts communicate in real time via a communication system. The simulator allows to monitor four different tasks: head positioning, mask positioning, positive pressure ventilation, and chest compression (see S1 Video for a demo vide The individuals pictured in S1 Video have provided written informed consent (as outlined in PLOS consent form) to publish their image alongside the manuscript.

### Hardware

**Fig 1:**
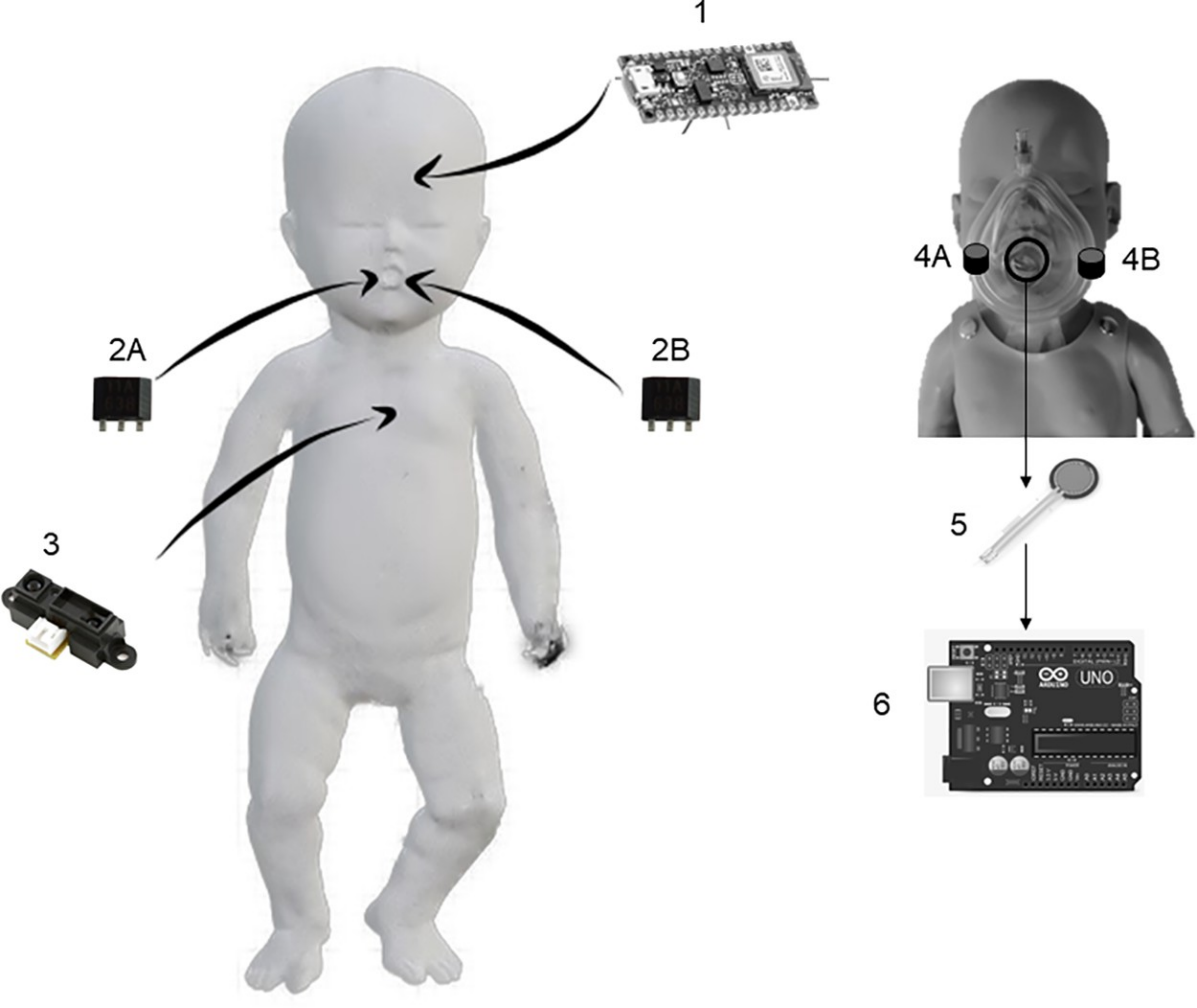
Hardware components. The hardware is composed by: (1) Arduino Nano 33 IoT with the IMU that monitors the orientation of the head; (2A) (2B) hall effect sensors which monitor the mask position; (3) IR sensor that allows to detect chest compression; (4A) (4B) magnets into the mask to recognize its correct positioning; (5) FSR used to monitor the positive pressure ventilation connected to (6) Arduino UNO REV3.

The hardware components of RiNeo MR include: a newborn manikin (M-1005745, 3b Scientific, Germany), having a single joint in the neck allowing the trainees to properly position the head during ventilation; two microcontrollers, an Arduino Uno REV3, and an Arduino Nano 33 IoT which integrates a 6-axis inertial measurement unit (IMU; LSM6DS3), that allows to monitor the orientation of the mannikin’s head; a force sensing resistor (FSR400; Interlink electronics, USA) sensor used to monitor the positive pressure ventilation; an infrared obstacle detection sensor (IR, GP2Y0A41SK0F; SHARP, Japan) to detect chest compression; two Hall effect sensors (SS443A; Honeywell, USA), which monitor the mask position; a Leap Motion (Ultraleap, USA) device to track the user’s hands in real time; an HTC Vive (HTC, Taiwan) system to immerse the trainee into the VR environment (Fig 1) (see S2 Appendix for further information on the sensors).

In detail, the IMU on the Arduino Nano 33 IOT board, located inside the manikin’s head (Fig 1) monitors the rotation of the head in the sagittal plane (antero-posterior) of the manikin’s head performed by the operator. Arduino provides the *Arduino_LSM6DS3* library that can be installed directly from the IDE to read the accelerometer and gyroscope data. To merge the accelerometer and gyroscope data, we used a library that contains the official implementation of the MadgwickAHRS algorithm, which allows for a more accurate estimation of the orientation of an object based on raw accelerometer and gyroscope data readings. Specifically, it returns the Euler Angles (Pitch, Roll, Yaw) that describe the orientation of a rigid body in space. Given that: (i) the manikin is always positioned on a stable horizontal plane, although not physically fastened to it; (ii) NLS requires placing the baby’s head and neck in a neutral position avoiding neck hyperextension and flexion; (iii) the joint in the neck allows movements only along the sagittal axis, the IMU monitors only antero-posterior rotations. The infrared sensor, that is a system consisting of an IR transmitter and IR receiver used to detect and calculate the distance to an object, is placed in the newborn’s back, at the sternum level (Fig 1), with the transmitter and the detector facing inside the manikin. Chest compression can be measured with several sensors (e.g., ultrasound, potentiometer, optical, potentiometer); however, our setup has strict space constraints, which require to use a small sensor that could be located on the manikin’s back and detect chest compression without being detectable by the user.

Moreover, two Hall effect sensors to detect the mask position are located under the dimples of the manikin’s lips and oriented in opposite directions (i.e., designed to respond to the north pole on one side and the south pole on the opposite side) (Fig 1). One sensor is activated by the presence of a positive magnetic field (south pole), the other one is activated by the influence of a negative magnetic field (north pole). In both cases, the output is deactivated if this field disappears or reaches a value below the activation threshold. To activate the sensors, the mask has been equipped with two permanent magnets arranged with opposite polarity. They are oriented and located so that both sensors are activated simultaneously when the mask is placed over the infant’s mouth in the correct position. Both sensors return a binary value, either a zero or a one, depending on whether the mask is correctly placed. In both cases, a "1" indicates correct coupling between the individual hall sensor and the corresponding magnet, while a "0" means either no magnet or wrong pole exposure.

**Fig 2:**
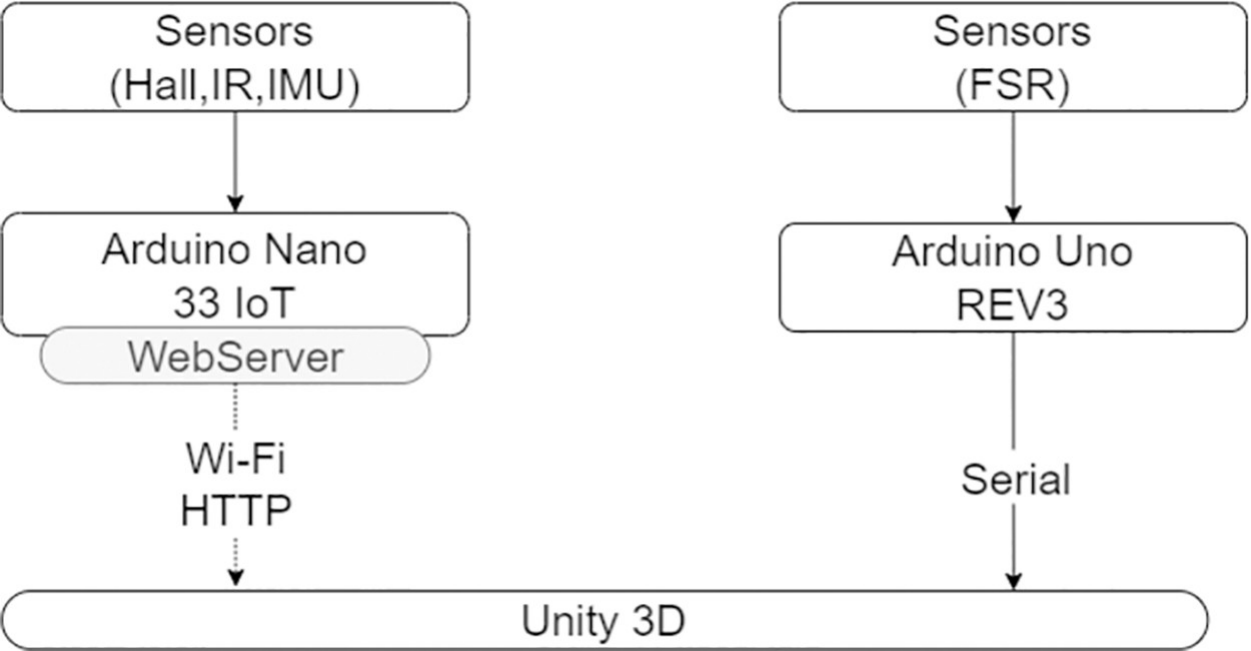
Communication system. Filled lines indicates serial communication, dotted lined show Wi-Fi communication.

All the sensors mentioned above (i.e., IMU, FSR and IR), are serially connected to the microcontroller Arduino Nano 33 IoT, powered by a power bank. This board receives data from sensors and sends it in real time via Wi-Fi to a computer running the application developed in Unity3D (Fig 2). Finally, Arduino UNO REV 3 acquires data from the Force Sensing Resistor sensor, connected via serial, which monitors how the user is performing the positive pressure ventilation. The sensor has been positioned at the ventilation inlet at the top of the T-piece cap, below a plastic membrane so as not to be perceived by the user. In this position, it can detect the occlusion of the gas leakage opening and, consequently, monitor the user’s performance of the ventilation tasks. For our project, the goal was to capture binary ON/OFF information, specifically whether the sensor was pressed or not, without the need to measure the applied force. Data from this board are sent via serial to the Unity 3D application (Fig 2). Finally, all data received by the computer from both the Arduino can be saved and stored for future evaluation, as well as to assess the trainees’ performance.

**Fig 3:**
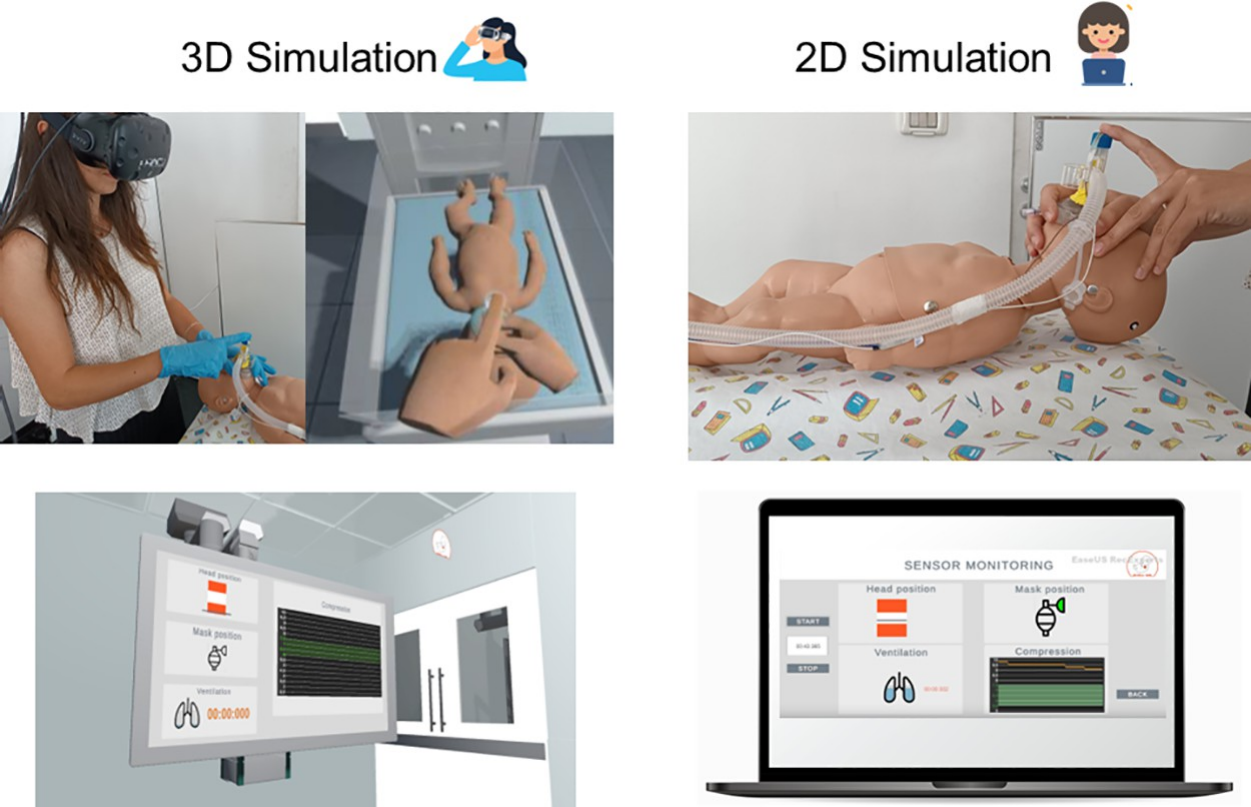
2D and 3D simulation. Left: 3D simulation. The user wears the HMD and is immersed into a virtual environment showing a virtual representation of the manikin. Thanks to an HTC controller that tracks the neonatal island position, the virtual and real manikins are overlapped to each other and move accordingly. Right: 2D simulation. The user can physically interact with the manikin and receives feedback from a User Interface (UI) displayed on a computer screen. Sensors in the manikin and the face mask sends data to the immersive VR application or the GUI to monitor the user performance in real time. UI description: Top left: the UI provides feedback about the manikin’s head position. Top right: mask position; simple controls have been implemented to manage the colour code; if the area turns red, completely incorrect positioning; if the mask turns yellow, partially incorrect positioning; if the area turns green, correct positioning. Bottom left: Ventilation rhythm If the sensor in the face mask is pressed (inhalation), the lungs in the UI are filled, during exhalation phase (sensor not touched), they are empty. Bottom right: chest compressions are shown on a line chart according to the data measured by the infrared sensor.

Other than sensors, the system includes an HTC Vive headset system, used both as a tracking system and to navigate the virtual environment. One controller is fixed into the neonatal island to monitor its position, and consequently the newborn position, as the manikin is constrained in the neonatal island (Fig 3). This way, movements of the physical neonatal resuscitation table are monitored in real time and reported in the virtual environment, as movements of the virtual table. This is particularly important, as the match between the real and the virtual manikin is crucial for the success of the simulation in the 3D mode. Indeed, in a previous study [26], the usability of the HTC Vive controller and tracker for a mixed reality medical simulator was assessed. Another tracker is attached to the face mask (Fig 3), to track its position. The system also includes a Leap Motion device mounted on the HTC Vive headset that tracks the user’s hands in real time and develop possible interactions within the environment. As data from the HTC Vive system, Leap Motion and sensors are managed by the VR application, there is no requirement for specific calibration phases between the systems.

### Software

RiNeo MR has been designed to be used in two different modalities: 2D and 3D (Fig 3). The former modality does not involve using the HTC Vive visor, as the performance is shown in real-time through the application’s two-dimensional interface on a computer screen (Fig 3). In the 3D mode, the user wears the VR headset being immersed into a VR scenario which receives data in real time from the sensors, to create the desired actions. The software has been developed in the Game Engine Unity3D via Steam VR.

To have a real time communication between the real and virtual worlds, namely the sensors located in the manikin and face mask and the 2D/3D Unity application, we have implemented a web server communication system and serial communication (Fig 2).

**Fig 4:**
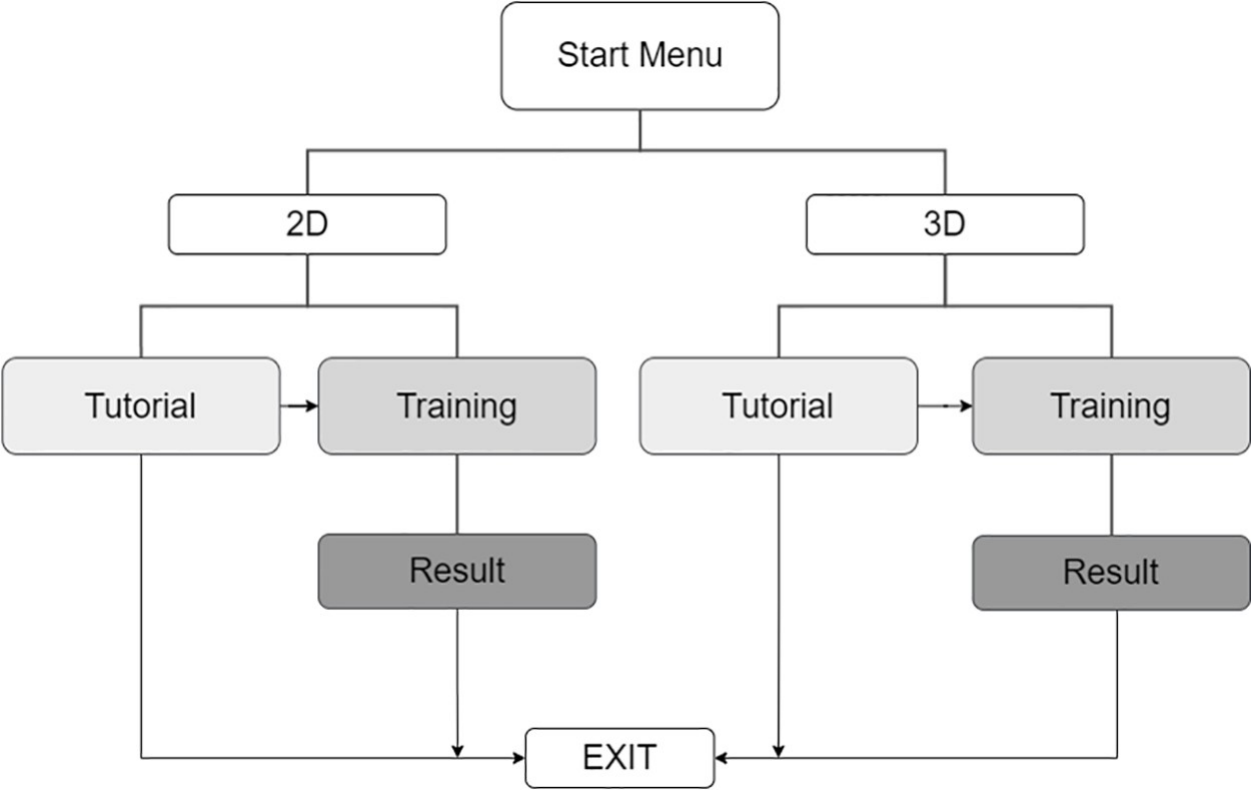
Scene. First button “Start Menu” allows the user to choose whether to proceed in 2D or 3D simulation mode. The user can then choose whether to view the tutorial (i.e., an explanation video) or enter the simulation training scene. After the training, it is possible to view the results obtained during the simulation. In every scene, there is the possibility to go back to the previous one (dotted lines).

The software architecture of RiNeo MR consists of 9 scenes organized as shown in Fig 4. The transition from one scene to another is managed by the user who interacts with different buttons. Scene selection can be done either by mouse click in the 2D scenes, or by using the user’s hand, tracked by the Leap Motion device, in 3D environments. Importantly, the mouse is only used in the 2D environment, to make scene selections (for instance, choosing between a tutorial or training scenario or beginning/pausing/ending the scenario). Then, the trainee interacts physically with the manikin (e.g., positioning its head, performing chest compression etc.). In the 3D environment, the same selections are performed using the hands, as the Leap Motion tracks their movements. Furthermore, the Leap Motion allows visualizing the hands in real time, thus guiding the user in the VR without the use of controllers that would limit hand movements during NLS. Importantly, no interactions are measured by collecting Leap motion data, but the trainee’s performance is measured using sensors in the manikin.

#### 2D mode

The goal of our study was to create a realistic simulation, without the need of a lifelike setting, However, as the system includes both a sensorized manikin and a VR application, we decided to let trainees and instructors use the manikin in the “traditional” way, namely without the immersive application, but only with a screen-based User Interface (UI). Typically, simulation-based training uses a manikin to simulate a specific manoeuvre. This can be particularly useful in the first stages of training, when the user wants to show a specific manoeuvre or behavior. In the 2D mode, the user can perform neonatal resuscitation manoeuvres and receive a real time feedback through a screen-based UI (Fig 3). This modality includes two options: "Tutorial" to watch a demonstration clip of neonatal resuscitation manoeuvres, such as manual stimulation, opening of the airway, positive pressure ventilation, and chest compressions; "Training" to start the simulation and practice on the physical manikin. During the training, the UI provides real time feedback about: (i) orientation of the newborn’s head, by showing the data coming from the IMU in the manikin’s head (Fig 3, UI top left). When the slider is in the orange areas, manikin’s head is either over-extended, or excessively flexed. If the slider is in the white area, then the correct neutral position has been reached. (ii) mask positioning (Fig 3, UI top right), according to the values of the magnetic sensor. In this case, simple controls have been implemented to manage the colour code of the mask icon: if the positioning is completely incorrect (i.e., none of the sensors detect the mask), the area turns red; if the mask in partially positioned, (i.e., one of the sensors detects the mask), the area is yellow; if both sensors properly detect the mask, the area turns green. (iii) ventilation rate, by detecting the users’ occlusion of the gas leakage opening at the ventilation inlet at the top of the T-piece cap through the FSR sensor (Fig 3, UI bottom left). Indeed, we have implemented a state machine, made up of two states. One state relates to the sensor pressure (inhalation) and the other to the absence of pressure/raised breath (exhalation phase). (iv) Chest compressions rate and depth, either performed using the 2-finger or 2-thumb techniques. These measured are displayed in the User Interface on a line chart (Fig 3, UI bottom right).

#### 3D mode

**Fig 5:**
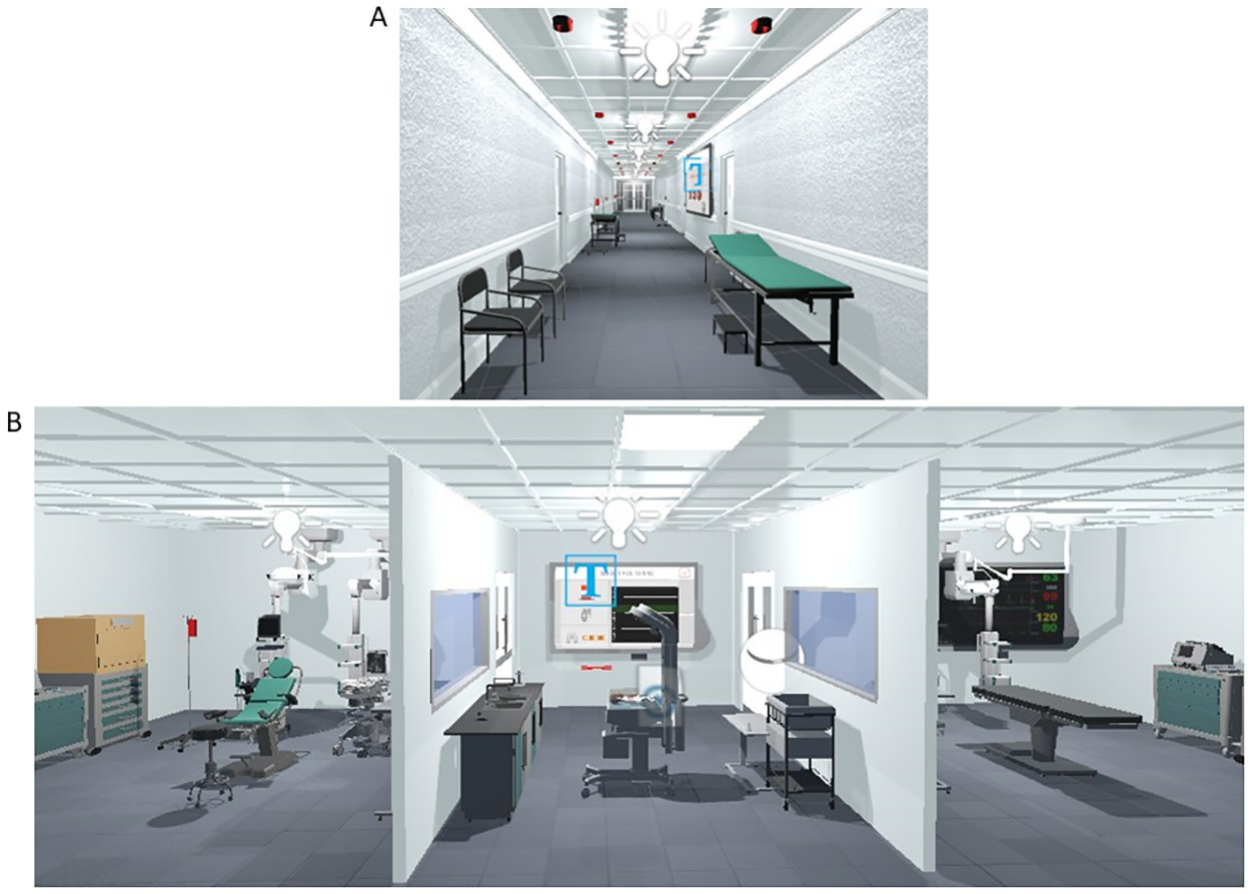
Virtual environment. (A) hospital hallway; (B) delivery room (bottom left), operating room (bottom right) resuscitation room (bottom middle). Inside the resuscitation room there is RiNeo MR user interface (UI).

In the 3D mode, RiNeo MR combines the functionalities of the 2D mode, with an immersive VR application available by using the HMD (Fig 3). Indeed, the trainee can watch the demonstration video (Tutorial) or proceed with the “Training”. With this configuration, the user is immersed into a virtual world where a medical emergency can occur and is able to practice NLS on a virtual manikin. As described in the hardware subsection, sensors and trackers positioned in the manikin, neonatal island, and face mask, monitor in real time the position of the manikin, other than the correct execution of resuscitation manoeuvres. This way, the real and virtual manikin are overlapped and behaves accordingly, thus guaranteeing a correspondence between what the user sees and what he touches, obtaining a coherent environment (Fig 3). At the current stage, the simulator offers to perform NLS in a hospital resuscitation room, that is located between a surgical theatre, as a deliver can occur spontaneously or be a result of a caesarean section, and a delivery room (Fig 5B; [31]). Also, prior to the simulation, the user can familiarize with the virtual environment, by being immersed in a hospital hallway (Fig 5A). All the rooms are furnished with realistic appliance (Fig 5) and have realistic dimensions, to immerse the user in an accurate setting [31]. Finally, starting from a 3D model of a newborn manikin, we have adjusted its size to those of the real one using Blender (Blender foundation, the Netherlands). This way, when the user interacts with the virtual manikin, for instance by placing the mask, the face of the virtual newborn has the same size and shape of the real one, otherwise the manoeuvre cannot be completed.

Like the 2D mode, the 3D contains a menu allowing the user to watch a Video Tutorial or to proceed with simulation training in the immersive virtual environment (Fig 5). As the user wears the HMD and is fully immersed in the virtual scene, in the 3D mode, real time feedback is provided both by a screen located in the resuscitation room (Fig 3). Also, we have added additional feedback by including animations associated to the manikin, such as chest expansion when the mask is properly positioned and ventilation is provided; head movements, according to the angles detected by the IMU in the manikin’s head.

### Tests

#### Subjects

After the design and implementation of RiNeo MR, we enrolled 16 subjects during the period June 2022 –July 2022: 11 people without medical expertise (age mean ± STD: 25.4 ± 2.1 years, 7 women) and 5 paediatric residents (age mean ± STD: 30.0 ± 0.7 years, 5 women) to test the simulator and collect feedback on its usability. Inclusion criteria were: no medical knowledge for the control group, and be part of a speciality school in paediatrics. The study was approved by the local Institutional Review Board (code CE DIBRIS protocol—010/2020 approved on 18/05/2020) and all subjects gave informed consent conforming to the ethical standards of the Declaration of Helsinki. Each participant gave their written, informed consent to take part in the study. The individual pictured in Fig 3 has provided written informed consent (as outlined in PLOS consent form) to publish their image alongside the manuscript.

#### Experimental design

**Fig 6:**
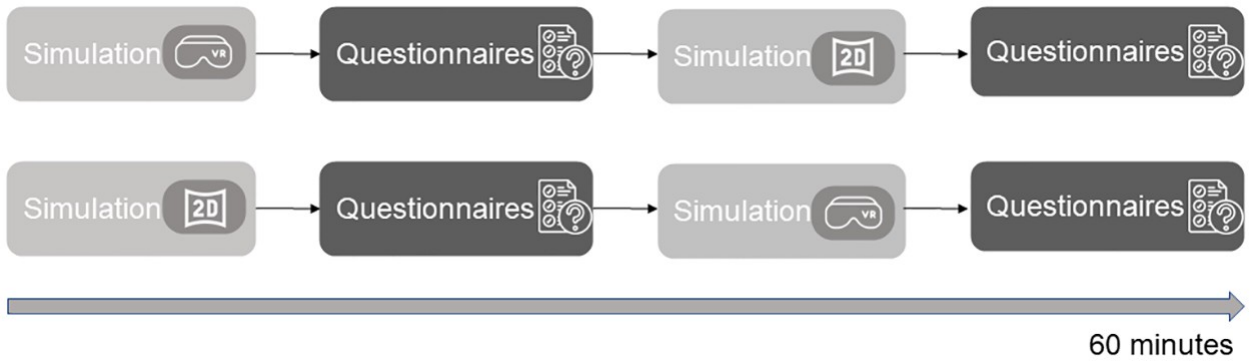
Pipeline of the experiment. First the subject randomly starts 2D or 3D simulation. After the first simulation, the subject fills out questionnaires to assess user experience, usability of the system, sense of presence and motion sickness. Then, he/she starts the second simulation, followed by additional questionnaires.

The experiment consisted of two phases: 2D simulation, and 3D simulation, followed by post-experiment questionnaires. The overall duration was of about 60 minutes divided as such: 15/20 minutes to fill out the questionnaire 15/20 minutes for the 2D mode, 25/30 minutes for the 3D mode, including the familiarization with the virtual environment. Participants started the experiment with 3D simulation or 2D simulation, randomly selected. During the session, subjects observed the elements of the simulation and interacted with them autonomously. At the end of each phase, users filled out questionnaires to evaluate the usability of the system, the sense of presence and whether any discomfort occurred (see below for more details, Fig 6). After the experiment, we interviewed the participants to collect feedback and opinions about the strengths and weaknesses of the system.

During the 2D simulation, subjects were asked to try neonatal resuscitation manoeuvres on the physical manikin; they included head positioning, mask positioning, ventilation, and chest compression, and to look at the screen reporting real time feedback on their performance (Fig 3). At the completion of this simulation, they completed two surveys: User Experience Questionnaire (UEQ; [32]), and System Usability Scale (SUS [33]; see below).

To perform the 3D simulation, the user wore the VR headset, and was immersed in the virtual hospital hallway (Fig 5A). As this scene was designed to let the subject familiarize with the virtual environment, he/she could move around, move their hands to see their virtual replica and interact with buttons in the scene (Fig 5). As soon as the participants felt at ease in the virtual environment, they pressed the button “Simulation” to start the session, which took place in the resuscitation room. Inside this second scenario, the user could perform several actions: (i) move the baby warmer; (ii) adjust the manikin’s head position; (iii) position the face mask and perform ventilation; (iv) compress the chest. Importantly, as the virtual and real manikin and baby warmer are overlapped, the user could perceive the real object, albeit immersed in the virtual scene. At the end of the 3D VR experience, we provided users with five questionnaires (see below): UEQ, SUS, Simulation Sickness Questionnaire (SSQ; [34]), and the Igroup Presence Questionnaire (IPQ; [35]).

#### Questionnaires

As mentioned above, we have selected different questionnaires to evaluate RiNeo MR in terms of: (i) usability, which is operationally defined as the user’s subjective experience when interacting with a system [36]; (ii) user experience, defined as the overall person’s experience with the system including design, graphics, interface, physical and manual interactions [37]; (iii) sense of presence, in terms of “being there” and perceive the virtual environment as real [35]; (iv) simulator sickness, or cybersickness, a subset of motion sickness that can be experience during VR experiences [38].

UEQ [32] covers a comprehensive impression of user experience, namely a collection of unique benchmarks that includes traditional usability standards like effectiveness, controllability, and learnability, as well as non-goal-directed or hedonic criteria like stimulation, fun-of-use, novelty, emotions, and aesthetics [39]. The questionnaire is composed by 26 items grouped into 6 scales: attractiveness, efficiency, perspicuity, dependability, originality, stimulation. Scales are not independent; in fact, a user’s general impression is captured by the attractiveness scale, that, in turn, is influenced by the values on the other 5 scales [40]. Perspicuity, efficiency, and dependability are pragmatic quality aspects (goal-directed), while stimulation and novelty are hedonic quality aspects (not goal-directed). Pragmatic quality describes task related quality aspects, hedonic quality is concerned with features that are not task-oriented, such as the user interface’s originality or aesthetic appeal [32]. Each UEQ item consists of a pair of terms with opposite meanings, and each item can be rated on a 7-point Likert scale [41]. Answer to an item therefore ranges from -3 (fully agree with negative term) to +3 (fully agree with positive term). Half of the items start with the positive term, the rest with the negative term (in randomized order) [40].

SUS [33] is a 10-item questionnaire, with five response options from Strongly agree to Strongly disagree, that provides an overall evaluation of usability. SUS items were created on the three usability criteria: (i) the capacity of participants to complete the tasks using the system and the quality tasks’ output (i.e., effectiveness); (ii) the amount of mental resources used to complete tasks (i.e., efficiency); (iii) the users’ subjective reactions to the system as a whole (i.e., satisfaction) [36]. To analyse this questionnaire, we have converted the scale as follows: strongly disagree 1 point; disagree 2 points; neutral 3 points; agree 4 points; strongly agree 5 points. Then, we have summed the points and multiplied the score by 2.5 to obtain a 1 to 100 scale. To interpret the data, we have transformed them into a percentile ranking: excellent (>80.3), good (68–80.3), okay (68), poor (51–68) or awful (<51) [42].

To measure the users’ level of sickness symptoms caused by virtual reality simulators, the SSQ [34] is frequently used. The questionnaire asks participants to score 16 symptoms on a four-point scale (0–3). Symptoms can be generally divided into three categories: Oculomotor, Disorientation, and Nausea [43]. We looked at each score, to assess whether a specific symptom has occurred during the simulation.

To determine the users’ sense of presence inside a virtual environment (VE) we used the IPQ [35], that is a 14-item, 7-point Likert scale questionnaire [44]. The IPQ has three subscales plus one general item not belonging to any subscale: (i) Spatial Presence, the sense of being in the VE; (ii) Involvement/Attention, measuring the attention devoted to the VE and the involvement experienced; (iii) Experienced Realism, the subjective experience of realism in the VE [45].

### Data analysis

Mean values and standard deviations have been computed for each item, score or subscore (see Data). Residents and controls data have been analysed separately to ensure that usability, user experience, sense of presence and simulator sickness are not affected by the ability in performing resuscitation manoeuvres. Group differences have been assessed using nonparametric Mann Whitney test.

## Results and discussions

### 2D simulation

**Fig 7:**
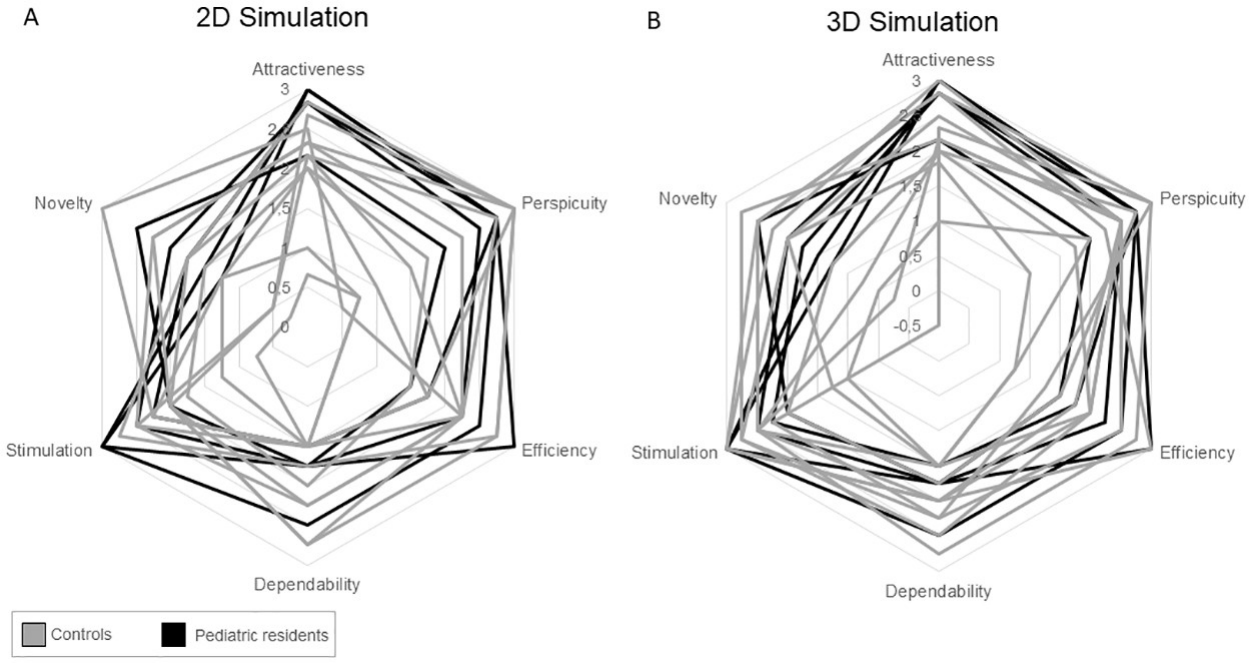
Results of the UEQ questionnaire. (A) Results of the 2D simulation; (B) Results of the 3D simulation. Grey lines indicate control subjects, black Paediatric residents. Scales range between -3 (negative) and +3 (positive).

Results of the UEQ questionnaire report values greater than 1 (range between -3 to +3) for all the six scales without differences between controls (C) and paediatric residents (PR), (Fig 7A; attractiveness, mean ± STD C: 2.1 ± 0.7, PR: 2.6 ± 0.4; perspicuity, C: 1.8 ± 1.0, PR: 2.0 ± 0.6; efficiency C: 1.9 ± 0.8, PR: 2.2 ± 0.8; dependability C: 1.9 ± 0.5, PR: 2.0 ± 0.6; stimulation C: 2.0 ± 0.6, PR: 2.0 ± 0.6; novelty C: 1.5 ± 0.9, PR: 1.2 ± 1.2), suggesting that user had a good user experience, while using the 2D version of the simulator.

**Fig 8:**
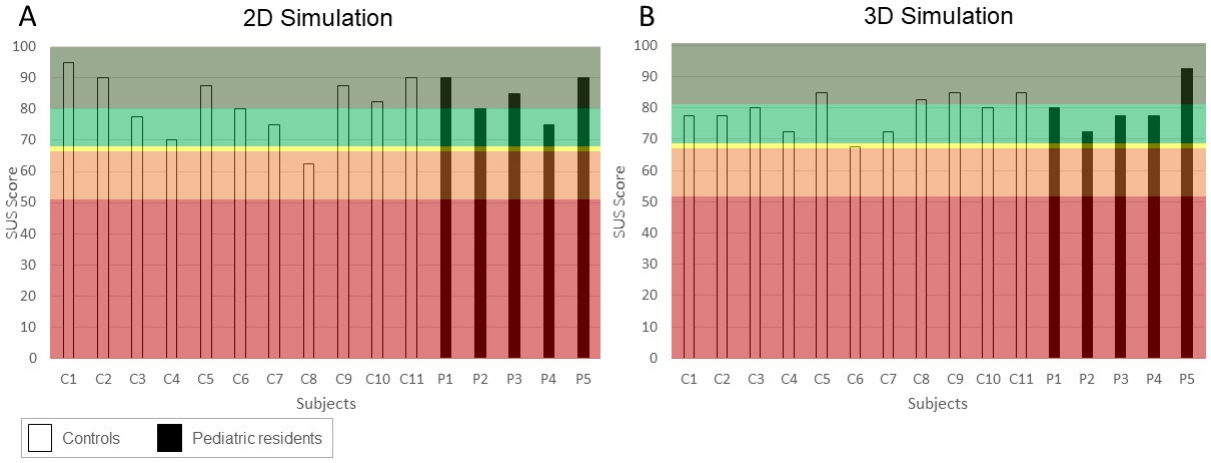
System usability scale scores. A. Results of the 2D simulation; B. Results of the 3D simulation. White bars indicate control subjects, black Paediatric residents. The dark and light green areas show excellent and good usability score. The yellow line indicates the threshold between neutral, poor (orange) and awful (brown) scores.

Fig 8A shows the SUS scores. As described above, scores greater than 68 indicate excellent or good ratings for usability. In general, all but one control subject reports an overall score equal or greater than 70, without differences between controls and paediatric residents.

### 3D simulation

Firstly, we have assessed whether the 3D version RiNeo MR caused any simulator sickness related to the use of VR., Analysis of the SSQ data did not reveal any discomfort; in fact, subjects reported levels of discomfort lower than or equal to 1 out of 3 for all symptoms listed.

As for the 2D simulation, results of the UEQ questionnaire report a good user experience level in all the scales (Fig 7B; attractiveness, mean ± STD C: 2.2 ± 0.6, PR: 2.8 ± 0.4; perspicuity, C: 2.3 ± 0.6, PR: 2.6 ± 0.3; efficiency C: 1.8 ± 0.7, PR: 2.2 ± 0.6; dependability C: 2.0 ± 0.4, PR: 1.9 ± 0.4; stimulation C: 2.3 ± 0.7, PR: 2.6 ± 0.5; novelty C: 1.5 ± 1.1, PR: 1.8 ± 0.5). In addition, SUS results indicate good to excellent usability scores for both groups, with only one subject rating the usability of RiNeo MR as poor (Fig 8B). Interestingly, in both 2D and 3D simulation, we obtained only one neutral to negative score by a control subject. One might think that the negative scores belong to the same subject. However, as visible in Fig 8, this was not the case. In fact, analysing single subject data, it has emerged that the negative score in the 3D simulation is due to the fact that the person feels he/she would need technical help to use the system. Conversely, in the 2D simulation the control subject giving a low score, would not use the system frequently.

Finally, IPQ did not show differences between the two groups (Table 1). Involvement and attention score, as well as experienced realism report mean values lower than 5 out of 7.

**Table 1:** IPQ results. Values (range 1–7) are shown as mean ± STD for each group (C: controls; PR paediatric residents). Involvement/Attention (INV); Experienced Realism (ER); Spatial Presence (SP); General Sense of Presence (PRES).

Group	INV	ER	SP	PRES
C	4.6 ± 0.9	3.4 ± 0.3	5.0 ± 0.6	6.0 ± 0.8
PR	4.4 ± 0.2	3.7 ± 0.5	4.9 ± 0.8	5.8 ± 0.8

Altogether, results revealed good levels of user experience and system usability. In particular, the results of the 3D UEQs showed an interesting result: we have found a trend toward significance (p = 0.067, Fig 7B) when we compared controls and paediatric residents’ attractiveness scores, with resident rating the attractiveness higher than controls. This difference could be explained by the fact that paediatric residents were able to understand the potential of RiNeo MR, in improving the NLS training of healthcare providers. Conversely, controls were immersed into a virtual world without having a clear vision of the educational potentiality and the final goal of the tool. This is further supported by the fact that, during the post-experiment interviews, some paediatric residents expressed interest in the use of the 3D version of RiNeo MR simulator and said they would be open to view and evaluate a future release. In addition, looking at Fig 7, paediatric residents, reported slightly higher scores also for novelty values. These results suggest that paediatric residents, and more generally medical students, have a good understanding of the difficulties related to medical learning and the benefits of using simulation techniques and technologies [46]. In fact, the practice provided by simulation training builds up confidence and hence satisfaction [47].

Finally, an interesting finding emerged from the analysis about the sense of presence in terms of perceiving the virtual environment as real. As mentioned above, involvement, spatial presence, and experienced realism report mean values lower than 5 out of 7, with no differences between the two groups. This can be explained by the fact that many subjects claimed that they felt the virtual world to be static and that they would have preferred to see more people and dynamic objects in the scene. The relationship between dynamic objects and sense of presence has been investigated in different ways [48, 49], suggesting that particular attention should be paid in the virtual environment, even if the main goal of the VR is to let trainees practice, rather than explore the virtual world. Another possible explanation relies on the fact that if on one hand the combination of virtual and real objects overlapped can enhance sense of presence, on the other hand, it can cause mismatches in the user perception, as some objects are both virtual and real (i.e., manikin, baby warmer), while others are not (e.g., walls, sink). Research on sense of presence in MR is still sparse [50]; thus, additional studies will be required to investigate whether sense of presence is different in MR with respect to VR.

With the increasing use of immersive technologies, numerous studies have been conducted to assess criticisms that could limit their use in the medical education setting. Even though immersive applications using HMDs are generally engaging and enjoyable [51], some studies highlighted issues, including discomfort due to the use of cumbersome equipment, difficulty of vision, motion sickness, and technical issues [51–53]. The latter embrace difficulties to start the application, move in the scenario, and interact with the objects [52]. Also, the face-to-face communication is limited, as most of the application are single player. This is especially relevant when instructor-based training is required. Another factor limiting the use of VR for medical training is the cost of the hardware (i.e., high-performance computers, and dedicated accessorized), as well as the need of dedicated personnel facing technical issues [52]. Nevertheless, constant efforts are made by companies and researchers to advance the technology and overcome these problems.

As mentioned above, the training of medical procedures is very important for medical students, residents, and healthcare providers. In fact, they all have different educational needs that can be achieved by using simulators [54]. For instance, medical students and residents need to learn manual skills [55] and established medical procedures, while clinicians and healthcare providers may need to refresh skills or to be update on new guidelines. In all these cases, training in an immersive environment combined with physical elements increases user’s engagement, immersivity and sense of presence [56], as also reported in our study, and produces better outcomes [57, 58].

## Conclusions

The goals of this project were to: (i) overcome the limitations of existing VR solution for medical simulation, namely the lack of passive haptic (that does not allow to simulate manual skills in a realistic way; (ii) design and develop a mixed reality system for newborn resuscitation training that could increase the number of healthcare providers able to perform high quality NLS; (iii) test the usability of the system.

Although the main manoeuvres for NLS training are monitored, additional steps (i.e., checking newborn temperature, advanced airway management, and umbilical line placing to let drugs administration) can be evaluated and implemented to further increase the educational potential of the system. This can be achieved by adding sensors both to the first aid supplies and the manikin.

The system, in its current form, uses commercial trackers and cameras to monitor the manikin’s position in the real world, and movements of the users’s hands. If, on one hand, these technologies are easy to use; on the other hand, they present limitations: (i) the tracking of the hands can be lost when they cross virtual objects; (ii) the trackers used are cumbersome, and, if touched, they may affect the overall experience. Indeed, given the fundamental importance of correct tracking and visualization of virtual hands [59], further research will be pursued to have tracking systems which are accurate and manageable.

Results from the questionnaire, as well as interviews with potential users revealed that the system is generally high rated for user experience and system usability, however; it has been reported that adding sounds and movements could further improve the realism and the immersivity of the application. These suggestions are further supported by the literature. Indeed, studies on how to improve realism in VR settings suggest working on the visualization techniques, such as global illumination, dynamic shadowing, ambient occlusion, and physically based rendering materials. Another possibility is to add avatars that move and react to users’ action, as this has been reported to enhance realism [60]. Therefore, we are planning to modify the lighting to improve scene shadows, incorporate materials with more accurate rendering, introduce animated avatars into the virtual environment, and add realistic elements to the scene (e.g., date and time information, posters and fliers commonly displayed in hospital settings).

Acknowledging the existence of other potential methods of training for newborn life support (NLS), it is important to mention augmented reality (AR) [61]. AR can be seen as a less immersive alternative to virtual reality (VR) as it combines virtual elements with the user’s actual surroundings without completely separating the user from the real world [62]. This reduces the full sensory immersion that VR provides and may result in a less focused and engaged training session, which was the main objective of our project. However, AR still has the potential for broader accessibility and the ability to integrate real-world interactions with virtual elements, offering valuable training opportunities, albeit different ones, especially in situations where full VR technology is not feasible [63].

Our study is a pilot study on the design and development of a system combining VR with a physical manikin for NLS training. Consequently, we have chosen to concentrate our efforts on usability and user experience, preliminary yet significant aspects. Nonetheless, we have plans to validate the RiNeo MR in an authentic NLS teaching environment, by conducting a comparison with other existing tools to determine its efficacy in both short-term and long-term learning outcomes. Given the pedagogical interest of our system, we will carry out a more extended study, involving a larger number of healthcare providers to assess the effectiveness of our simulator compared to manikin-based trainings. This might include investigating whether the manoeuvres execution speed and precision of the healthcare providers in the mixed reality environment are comparable to those performed on manikins or real practice. In this usability study we did not assess gesture speed or precision, as participant pool included individuals without a medical background who would naturally perform movements at a slower pace.

In conclusion, RiNeo MR is a proof of concept of a mixed-reality NLS simulator. The system can be a promising tool to improve NLS training and spread newborn resuscitation knowledge among all staff involved in perinatal medicine.

## Supporting information

S1 Appendix Newborn life support algorithm. This supplementary material provides additional information about the NLS Algorithm.
(DOCX)

S2 Appendix Sensors. This supplementary material provides comprehensive technical specifications of the sensors employed in our simulator setup.
(DOCX)

S1 Video. RiNeo MR DEMO. This video shows a practical DEMO about how the simulator works and how it is structured (MP4)

S1 Data (ZIP)
