# Supplementary material for: RiNeo MR: A mixed reality simulator for newborn life support training
Source: PLoS One. 2023 Dec 21;18(12):e0294914. doi: 10.1371/journal.pone.0294914 (PMC10734996; doi:10.1371/journal.pone.0294914)
Supplement: S1 Appendix — This supplementary material provides additional information about the NLS Algorithm. (DOCX) [file pone.0294914.s001.docx]

## **S1 Appendix. Newborn Life Support Algorithm**

Neonatal resuscitation is defined as the set of interventions needed at birth to support the establishment of respiration and circulation in the newborn [1]. The Neonatal Resuscitation Program (NRP) algorithm describes the resuscitative procedures required at birth. According to the International Liaison Committee on Resuscitation (ILCOR) 2020 guidelines [2], the NRP algorithm can be divided into five blocks from the moment of conception: Initial assessment, Airway (A), Breathing (B), Circulation (C), and Drug (D) [1]. During the initial assessment, it is required to determine whether the infant is healthy. If not, the baby should be placed in the neonatal island under a radiant heater and receive one or more additional interventions, the basic one is manual stimulation of the back/feet. The A phase aims at stabilizing a non-vigorous preterm newborn (i.e., establish an open airway and support spontaneous breathing) [1]. These previous actions often provide sufficient stimulus to start breathing. Otherwise, positive pressure ventilation (PPV) may be required. Approximately 60 seconds are allotted for completing the initial steps and beginning ventilation (block B). Ventilation is the most important and most effective action during neonatal resuscitation. PPV should be started if the infant is not breathing, or the heart rate is less than 100 beats per minute (bpm). To perform assisted ventilation, it is required to place the baby's head and neck in a neutral position, avoiding neck hyperextension and flexion. In addition, it is essential to choose the correct size of the mask and position it correctly on the newborn's face to avoid air leaks. A breathing act is generated by using a T-piece device, with a finger to alternately occlude and open the gas escape opening [1,3]. During assisted ventilation, sets of 5 breaths are repeated, with chest expansion checked at each insufflation. The ventilation rate is 40-60 breaths per minute. After about 15 seconds of ventilation, the manoeuvre should be stopped, and a first assessment should be made. If the heart rate is below 60 bpm, perform corrective ventilation steps (i.e, mask adjustment, head repositioning, suction, mouth opening, pressure increasing, use of tracheal tube or laryngeal maks) [1], before starting chest compressions (block C). Chest compression can be performed in two ways: compressions with two fingers placed perpendicularly on the sternum (2-finger technique), or by surrounding the baby’s chest with both hands and using the thumbs (2-thumb technique). In both cases, the correct area to perform chest compressions is below the imaginary line that joins the nipples, in the lower half of the sternum. The depth of compression should be approximately 3-4 cm [1], and the ratio of compressions-ventilations must be 90 compression per minute [4]. After about 60 seconds of ventilation and chest compressions, the heart rate should be re-evaluated. If it remains below 60 bpm, adrenaline should be administered (block D). Resuscitation should be stopped if, after 10 minutes of correctly performed manoeuvres, there is no heartbeat and no respiratory sounds, according to the global clinical evaluation of the patient.

1. Weiner GM, Zaichkin J, Kattwinkel J. Textbook of neonatal resuscitation. American Academy of pediatrics Elk Grove Village, IL; 2016.

2. Nolan JP, Maconochie I, Soar J, Olasveengen TM, Greif R, Wyckoff MH, et al. Executive summary: 2020 international consensus on cardiopulmonary resuscitation and emergency cardiovascular care science with treatment recommendations. Circulation. 2020;142: S2–S27.

3. Care EC. Circulation Part 5 : Neonatal Resuscitation Resuscitation and Emergency Cardiovascular Care. 2020;142.

4. Wyckoff MH, Aziz K, Escobedo MB, Kapadia VS, Kattwinkel J, Perlman JM, et al. Part 13: Neonatal resuscitation: 2015 American Heart Association guidelines update for cardiopulmonary resuscitation and emergency cardiovascular care. Circulation. 2015;132: S543–S560.
