# Supplementary material for: RiNeo MR: A mixed reality simulator for newborn life support training
Source: PLoS One. 2023 Dec 21;18(12):e0294914. doi: 10.1371/journal.pone.0294914 (PMC10734996; doi:10.1371/journal.pone.0294914)
Supplement: S2 Appendix — This supplementary material provides comprehensive technical specifications of the sensors employed in our simulator setup. (DOCX) [file pone.0294914.s002.docx]

## **S3 Appendix. Sensors**

In this supplementary document, we provide comprehensive technical specifications of the sensors employed in our simulator setup.

To monitor the orientation of the manikin's head, we selected the LSM6DS3 as inertial measurement unit (IMU). This component offers a combination of small size, affordability, and high performance. It is integrated into the Arduino Nano 33 IoT board and is available in a compact plastic land grid array package measuring 2.5 x 3.0 x 0.83 mm, making it suitable for ultra-compact solutions. Specifically, the LSM6DS3 is a 6-axis IMU equipped with a digital accelerometer and gyroscope, both of which are 3-axis. It is constructed using MEMS (Micro-Electro-Mechanical Systems) technology, which integrates electronics and mechanics into a single chip.

To monitor positive pressure ventilation using force sensing resistor (FSR) sensors, we employed the FSR400 model (Interlink electronics, USA). Resistive sensors convert variations in the input quantity into changes in resistance exhibited at their terminals. For our project, the goal was to capture binary ON/OFF information, specifically whether the sensor was pressed or not, without the need to measure the applied force. The FSR400 is a single-zone Force Sensing Resistor with a circular active area measuring 5.1mm in diameter and a length of 6 cm. This sensor is designed to detect force within a range of 100 g to 10 kg with actuation Force 0.1N and sensitivity 10N.

We used two Hall effect sensors SS443A (Honeywell, USA), to monitor the position of the mask. These sensors are compact, cost-effective, unipolar, digital magnetic sensors with a 3-pin configuration (power, ground, signal). They operate within a voltage range of 3.8 V to 30 V and are engineered to detect the presence of a magnetic field. Notably, as the sensors are oriented in opposite directions, one of them is responsive to the presence of a north pole magnetic field, while the other sensor reacts to a south pole magnetic field.

To detect chest compression, we selected an infrared obstacle detection sensor, specifically the SHARP GP2Y0A41SK0F model (SHARP, Japan). In our project, we utilized the SHARP 0A41SK F71 infrared sensor, which has dimensions of 29.5 x 13 x 13.5 mm. This sensor is capable of measuring distances ranging from 4 to 30 cm when supplied with a voltage between 4.5 and 5.5 V. It takes measurements at intervals of 16.5 ms. The output distance characteristics are represented graphically, showing that the output voltage varies from 2.7 V when an object is at a proximity of 4 cm to 0.45 V when an object is at a distance of 30 cm.
